# Structural Plasticity and Conformational Transitions of HIV Envelope Glycoprotein gp120

**DOI:** 10.1371/journal.pone.0052170

**Published:** 2012-12-27

**Authors:** Anil Korkut, Wayne A. Hendrickson

**Affiliations:** 1 Department of Biochemistry and Molecular Biophysics, Columbia University, New York, New York, United States of America; 2 Department of Physiology and Cellular Biophysics, Columbia University, New York, New York, United States of America; 3 Howard Hughes Medical Institute, Columbia University, New York, New York, United States of America; George Mason University, United States of America

## Abstract

HIV envelope glycoproteins undergo large-scale conformational changes as they interact with cellular receptors to cause the fusion of viral and cellular membranes that permits viral entry to infect targeted cells. Conformational dynamics in HIV gp120 are also important in masking conserved receptor epitopes from being detected for effective neutralization by the human immune system. Crystal structures of HIV gp120 and its complexes with receptors and antibody fragments provide high-resolution pictures of selected conformational states accessible to gp120. Here we describe systematic computational analyses of HIV gp120 plasticity in such complexes with CD4 binding fragments, CD4 mimetic proteins, and various antibody fragments. We used three computational approaches: an isotropic elastic network analysis of conformational plasticity, a full atomic normal mode analysis, and simulation of conformational transitions with our coarse-grained virtual atom molecular mechanics (VAMM) potential function. We observe collective sub-domain motions about hinge points that coordinate those motions, correlated local fluctuations at the interfacial cavity formed when gp120 binds to CD4, and concerted changes in structural elements that form at the CD4 interface during large-scale conformational transitions to the CD4-bound state from the deformed states of gp120 in certain antibody complexes.

## Introduction

Human immunodeficiency virus HIV-1 is the etiological agent of acquired immunodeficiency syndrome (AIDS), which infects CD4^+^ lymphocytes in humans [1], [2]. The entry of HIV into target cells initiates with the sequential interaction of gp120 subunits of viral envelope glycoprotein (Env) with CD4 glycoprotein receptor and the seven-transmembrane chemokine receptor on the host cell surface [3], [4], [5], [6]. Interaction of gp120 with its cellular receptors causes large conformational changes on gp120 as shown by biophysical, biochemical and crystallographic studies [7], [8], [9]. Such conformational changes are mainly induced by CD4 binding and are required to start the cascade of events leading to the fusion of viral and host membranes.

**Figure 1:**
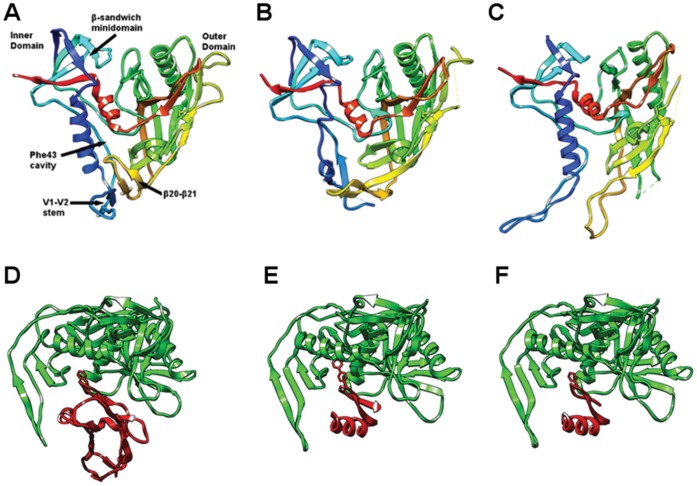
Structure of gp120 in different ligation states. (**A–C**) Comparisons of gp120 structures in (**A**) the CD4-bound state (PDB ID: 1G9N) [10], (**B**) the b12 antibody-bound state (PDB ID: 2NY7) [17], and (**C**) the F105 antibody-bound state (PDB ID: 3HI1) [20]. Structures are rendered as ribbon diagrams colored spectrally along the sequence from blue at the N-termini to red at the C-termini. The view is into the face occupied by CD4 interactions. (**D–E**) Comparisons of complexes between gp120 and various protein ligands: gp120 with (**D**) CD4 (PDB ID: 1G9N) [10]. (**E**) CD4M33 (PDB ID:1YYL) [16] and (**F**) F23 (PDB ID: 1YYM) [16]. Structures are rendered as ribbon diagrams with gp120 molecules in green and CD4 or its mimetics in red. The view has gp120 molecules rotated by ∼90° about the horizontal axis relative to the upper set (**A–C**).

The exterior envelope glycoprotein gp120 and the transmembrane protein gp41 together form the trimeric HIV protein Env on the virion surface [1]. The gp120 monomer is composed of five constant regions (C1–C5) interspersed with 5 variable regions (V1–V5). Crystal structures of complexes formed between the gp120 core, the membrane-distal immunoglobin (Ig) domains D1 and D2 of CD4 and an Fab fragment of antibody 17b provided the initial information on the structural basis of HIV entry to host cells [10], [11] (Figure 1A). The crystal structures revealed that the constant regions of gp120 fold into a core structure, whereas all the variable regions form loops that are bracketed by disulfide bridges with the exception of V5. As seen in the crystal structures, core gp120 lacks the variable loops V1, V2, V3 and the 85 residues from the C and N termini but it keeps its structural integrity and ligand binding ability as directly shown by calorimetric titration experiments [8], [12].

**Figure 2:**
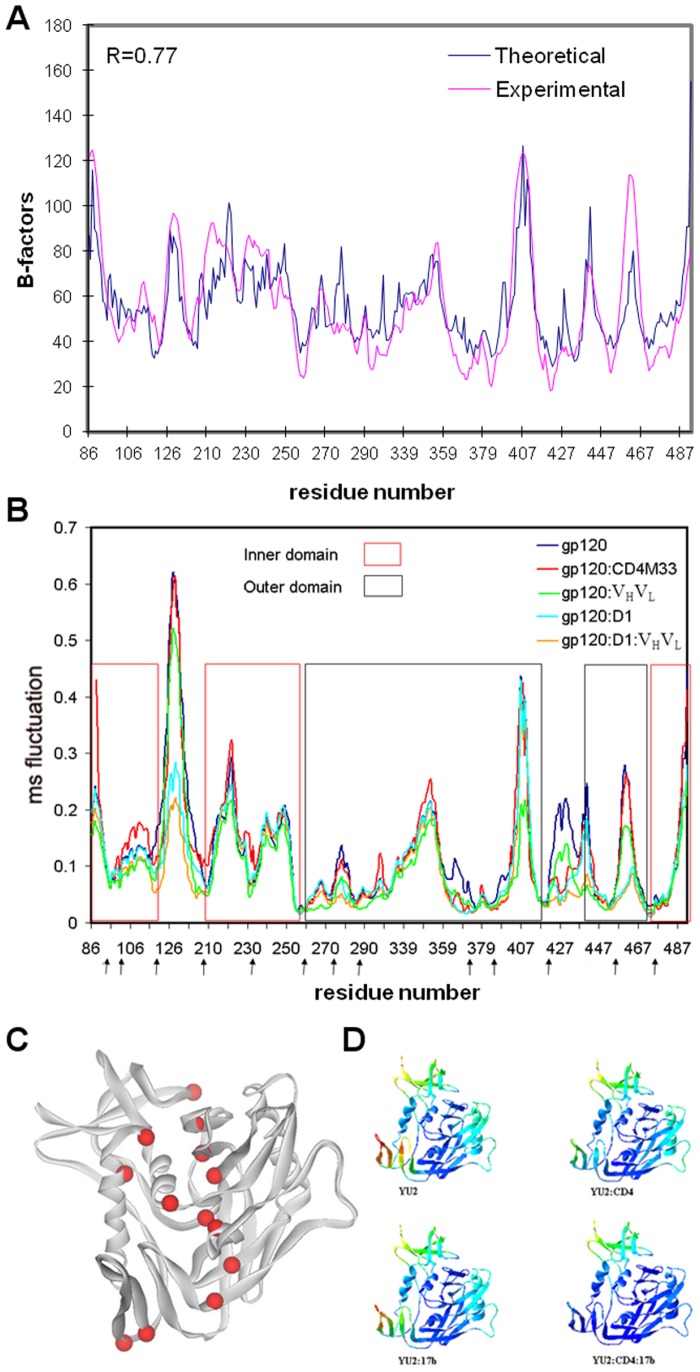
Collective motions in gp120 as computed by the ENM. (**A**) Comparison of experimental and ‘theoretical’ B-factors (mean-square fluctuations) for the structure of core HIV-1 gp120:CD4(D1D2):Fab17b. Experimental B-factors are from the crystal structure (PDB ID: 1G9N) [10]; theoretical values derive from the mean-squared fluctuations, <|**r** – **r_0_**|^2^>, as summed from all modes from an isotropic ENM calculation from the entire gp120:D1:V_H_V_L_ complex, noting that B = 8π^2^<|**r** – **r_0_**|^2^>/3. Thus, the ENM profile has been uniformly scaled to match the experimental data. The correlation coefficient between the two profiles, which is scale invariant, is 0.77. (**B**) Mean-square (ms) fluctuations represented by the frequency weighted sum of the 10 slowest modes. Analyses are from gp120 as isolated from the gp120:CD4∶17b complex and in various complexes as indicated in the color key. Arrows below the sequence place the hinge sites as determined from the analysis of gp120 in complex with the D1 domain of CD4 and the V_H_V_L_ domains of antibody 17b. (**C**) The hinge sites (red spheres) mapped onto a ribbon drawing of the gp120 structure (grey). (**D**) Ribbon diagrams of the gp120 structure color-coded spectrally according to residue fluctuations. Red corresponds to the highest amplitudes and blue to the lowest amplitudes.

In the liganded state, the structure folds into three major domains that are called inner domain, outer domain and the bridging sheet. The inner domain, which includes the N and C termini of the protein, consists of a two-helix, two-strand bundle at the termini-distal end and a five-stranded β-sandwich at its termini-proximal end. The outer domain is composed of a stacked double barrel, whose axis lies parallel to the axis of the inner domain bundle. The double barrel of the outer domain consists of a proximal mixed directional β-sheet that is twisted to embrace an α-helix as the seventh barrel stave and a distal barrel that is a seven-stranded anti-parallel β-barrel. The two barrels share one contiguous hydrophobic core and staves that continue from one barrel to the other are located at the inner-outer domain interface. The distal end of the outer domain includes an excursion via loop L_F_ into a β-hairpin, β20–β21, which in turn hydrogen-bonds to β2–β3 from the inner domain. The resulting four-stranded antiparallel β-sheet (β2–β3 and β20–β21) links the outer and inner domains to form a third domain, the bridging sheet. Note that we follow the secondary structure numbering annotated in the original description of the gp120 crystal structure, i.e. Figure 2A in reference [11].

In the crystal structures with gp120, CD4 binds to a depression formed by the interfaces of inner, outer and bridging sheet domains of gp120. The most critical CD4 binding region is a roughly spherical cavity, which is positioned at the intersection of all three domains of gp120 and capped by the Phe43 residue of CD4; hence it is called the Phe43 cavity. The Phe43 cavity extends into the hydrophobic core of gp120 and is lined by mainly hydrophobic and conserved residues. The mutations of residues such as Trp427 and Thr257 that line this cavity usually reduce binding of CD4 although they have limited contact with CD4 [13]. Factors such as the location of the cavity, the conserved amino acid residues lining the cavity and finally its direct interaction with CD4 Phe43 suggest that the formation of the stable Phe43 cavity is a crucial part of CD4 induced gp120 conformation [14], [15].

The crystal structures of gp120 in complex with the 17b antibody and scorpion-toxin (scyllatoxin) mimics of CD4, CD4M33 and F23 have also been reported [16] (Figures 1C–E). Scyllatoxin is a short peptide onto which the gp120 binding surface of CD4 was installed. In CD4M33, the Phe43 of CD4 is replaced by a biphenylalanine residue, which penetrates into the Phe43 cavity. In F23, the Phe43 is preserved as in CD4. Both mimetic molecules display lower binding entropies to gp120 compared to CD4. F23 shows a lower binding affinity but a higher neutralization breadth compared to CD4M33. Despite these thermodynamic and virological differences, they both induce conformations similar to that seen in the gp120∶CD4∶17b ternary complex.

The crystal structure of gp120 in complex with a broadly neutralizing CD4-binding-site (CD4bs) antibody b12 was reported at 2.3 Å resolution by Kwong and colleagues [17] (Figure 1B). In comparison to the CD4-bound state, significant rearrangements on the inner and bridging-sheet domains of the b12-bound state are observed. In the b12-bound state, the β2–β3 and β20–β21 strands of the bridging sheet detach from each other and the α1 helix of the inner domain goes through a helix-to-coil transition. The observed rearrangements in gp120 provide a direct evidence of large scale conformational transitions, which play important roles in both viral entry and resistance to neutralization by antibodies. Broadly neutralizing CD4bs antibodies such as VRC01 attach to gp120 through conformationally invariant CD4-binding sites on the outer domain and subsequently overcome the conformational barrier that diminishes the neutralization potency of most CD4bs antibodies [18]; however, the structure of gp120 in complex with NIH45–46, a clonal variant of VRC01, reveal that additional antibody contact sites on inner domain and bridging sheet further increase the potency of CD4bs antibodies [19]. The role of structural changes in immune evasion and viral entry are further supported by other structures, in which gp120 is in complex with two poorly neutralizing CD4bs antibodies, F105 and b13 [20]. A detailed structural comparison of gp120 conformations lead to the suggestion of a “layered” gp120 architecture with structurally invariant β-sandwich and plastic inner-domain layers that facilitate the gp120 conformational transitions required for viral entry and immune evasion [21].

In addition to crystal structures of gp120, a series of experiments have shown the role of structural plasticity and large conformational changes in viral entry and adaptation to host immune responses. Large entropy barriers for ligand binding measured by isothermal titration calorimeter [8], [12], [16], mutational analysis of the critical sites [15], [22], existence of a glycan shield to evade immune responses [23], observation of conformational changes by CD spectroscopy [9], and demonstration of an equilibrium between distinct conformational states by measuring the binding of a panel of conformation specific antibodies to structurally restrained gp120 in solution [24] all provide evidence on the existence of such conformational changes and structural plasticity. The experimental evidences of the structural plasticity of gp120 increase the necessity for an analysis of gp120 dynamics in various different states and in atomic detail.

A few previous computational studies have addressed the near native state dynamics of gp120 with a 10 ns MD simulation [25], a graph-theory based method [26] and by a steered MD simulation, where motions of the bridging sheet upon force application have been analyzed [27]. More recently, a communication network of residues within the gp120 core domains was revealed with a computational fluctuation analysis [28]. In another study, a molecular dynamics analysis suggested that the binding of small molecule NBD-556 to Phe43 cavity enhanced the mobility of the outer domain [29]. However, an integrated computational analysis of gp120 structural plasticity and, particularly, the computation of large scale conformational transition pathway of gp120 are lacking. A systematic computational analysis of gp120 that spans a wide range of dynamics events at large temporal and spatial spectrum will enable us to better understand the HIV entry mechanism, effects of ligand binding on gp120 dynamics and sites of gp120 that are vulnerable to external perturbations such as therapeutic interventions to inhibit viral entry.

In order to compute realistic conformational transition pathways of protein molecules, we have developed a virtual atom molecular mechanics (VAMM) force field [30] and a related algorithm to compute conformational transition pathways [31]. The application of the VAMM based algorithm to adenylate kinase (ADK) and its comparison to simple harmonic potentials have proved that the method generates realistic transition pathways and that it generates intermediate structures more efficiently than other coarse grained methods. The VAMM algorithm uses an iterative normal mode calculation approach combined with energy minimizations to generate intermediate structures using two end states of a given protein to converge to a common intermediate state. The normal modes that contribute most to the transition are selected to generate a trajectory at each step of the transition, performing all calculations with the VAMM force field.

Here, we have undertaken a thorough and systematic computational analysis of gp120 structural plasticity and conformational transitions in order to gain further insights into the molecular mechanisms of conformational changes on HIV gp120 and its complexes with CD4, CD4 mimics and antibody 17b. Our computational analysis reveals the dynamic properties of gp120 including local fluctuations at the Phe43 cavity, the nature of collective domain motions and the hinge sites that coordinate those motions, the influence of ligands on gp120 plasticity, and finally large-scale conformational transitions that drive the major rearrangements of the molecule. First, a coarse-grained isotropic elastic network approach [32], [33], which uses a simple harmonic potential is applied to compare the fluctuation dynamics of gp120 in various ligation states and determine the location of global hinges. Secondly, a more detailed approach is taken to further analyze the structural plasticity of gp120 and its domain motions. For this purpose, a full atomic normal mode analysis (NMA) [34] in a solvent shell [35], which is equilibrated by a molecular dynamics simulation, is performed using the CHARMM simulation package and CHARMM22 force field parameter set [36]. This NMA analysis gives descriptions both for the collective domain motions of gp120 and also for local fluctuations at the conserved residues lining the Phe43 cavity. Finally, the conformational transition of gp120 and the motions of gp120 away from its native states are computed using the VAMM-based conformational transition algorithm.

## Results

### Isotropic Elastic Network Analysis of Ligand Binding and Structural Plasticity

Elastic network models inspect the local packing density and coordination of each amino acid residue to determine the range of motions available in the folded state. The distribution of motions induced by individual modes (also called mode shapes) can be predicted using an Elastic Network Model (ENM) [32], [33], [37]. In such models, the slowest modes (global modes) usually provide information on motions relevant to function. Here, we compare the “intrinsic flexibilities” of gp120 in the apo form and in complexes with CD4, 17b and CD4M33 using the isotropic ENM approach, which is also called the Gaussian Network Model [32], [33]. A generalization that introduced directionalities for oriented fluctuations was called the Anisotropic Network Model [37]; we use ENM as an umbrella term covering both the isotropic [32], [33] and anisotropic [37] versions.

One major problem associated with comparing the slowest modes of motions between the isolated gp120 and its complexes is filtering out the possible hinge bending motions that belong solely to D1D2 and 17b protomers. For this purpose, only the D1 domain of CD4 is used so that all gp120-CD4 interaction sites are retained during the calculations and the potential hinge bending motions of D1–D2 domains are filtered out. A similar approach is taken for the 17b molecule such that only the V_H_ and V_L_ domains of the 17b antibody are used in the ENM calculations. The global mode shapes are calculated individually for gp120, gp120:D1, gp120:CD4M33, gp120:V_H_V_L_ and gp120:D1:V_H_V_L_, each as isolated from crystal structures of complexes with the aim of understanding the changes in the intramolecular dynamics of gp120 upon receptor and antibody binding. In the case of gp120: V_H_V_L_ and gp120:D1:V_H_V_L_, an ultraslow ENM mode is observed. Comparison of a full atomic NMA and anisotropic ENM calculations (data not shown) indicates that this mode corresponds to the rigid-body motions of the 17b antibody along the gp120 interaction surface in the gp120:V_H_V_L_ and the ternary complex. Thus, we eliminate the 1st slowest mode from the analysis of gp120:V_H_V_L_ and the ternary complex to display the intramolecular plasticity in gp120. To compare gp120 plasticity in various complexes, the 10 slowest modes are frequency-weighted, summed up for each state and the resultant combined modes are compared between different complexes of gp120.

#### ENM-generated atomic mobility factors correlate with experimental values

In order to test the validity of the ENM calculations, we compare the ENM generated atomic mobility factors (B-factors) of gp120 complex with those experimentally observed in gp120 crystal structure (pdb ID: 1G9N, YU2 strain) [10]. We compute the theoretical B-factors as the frequency weighted sum of all fluctuation vectors [32]. The theoretical B-factors capture all the features observed in experimental B-factor profile over all gp120 residues (Figure 2A). The correlation coefficient between the theoretical and calculated values is 0.77, which indicates a highly quantitative correspondence. Thus, ENM can reproduce the experimentally observed local fluctuations of gp120 residues. This validates the use of the ENM for computing gp120 plasticity through analysis of individual slow modes of motion, which capture collective motions of gp120 (sub)domains.

#### Ligand binding reduces structural plasticity at the bridging sheet

ENM calculations reveal that mode shapes of gp120 in the isolated state and in complexes with CD4, CD4M33 and 17b are almost identical except for the bridging sheet (Figures 2B, D). The mean-square fluctuations around the bridging sheet are highest in the isolated state of gp120, indicating a high degree of mobility in this region when no receptor molecule is bound to gp120. When gp120 is bound to CD4, some degree of decrease in mobility is observed at sites that interact with CD4 but the most drastic effects are observed around the bridging sheet. Binding of CD4 to gp120 significantly depresses the mobility of residues on the bridging sheet. The formation of the ternary complex further lowers the flexibility of the bridging sheet. Also, when the bridging sheet is complexed only to the 17b antibody, it possesses intermediate fluctuation amplitudes as compared to isolated and CD4-bound states. CD4M33 does not show any significant effect on the β2 and β3 strands. However, CD4M33 and CD4 have similar effect on β20 and β21 of the bridging sheet showing that this site has been significantly stabilized when gp120 is bound to its ligands. The depression of fluctuations around the β20 and β21 is expected since CD4M33 interacts extensively with these strands of the bridging sheet. ENM analysis of gp120 in complex with F23 yields mode profiles identical to those of gp120:CD4M33 (data not shown).

This gradual decrease in the fluctuations of the bridging sheet upon receptor binding indicates that both CD4 and chemokine receptor binding contribute to stabilization of the fusion-state conformation of gp120, assuming that the 17b antibody mimics the effect of chemokine receptor on gp120. CD4M33 has an intermediate effect on gp120 such that only the β20 and β21 strands of the bridging sheet are affected by CD4M33 binding. The gradual decrease in the mobility of the bridging sheet upon binding of receptor and antibody is in accordance with the experimental results obtained from isothermal calorimetry measurements for the binding of CD4, CD4M33 and 17b to gp120 [16].

Even though the amplitudes of fluctuations significantly differ for the different states of gp120, especially around the bridging sheet, the shapes of the fluctuation profiles are mostly conserved between all of the complexed and isolated states. However, even with a low-resolution computational model such as ENM, critical local variations between the mode profiles related to different ligation states are observed. Notably, Gln428, which is located on the loop between β20 and β21, is at a depression in the mode profiles of all gp120 states including the CD4 free states. This suggests that Gln428 enjoys a lower degree of mobility and a lower entropic barrier for CD4 binding compared to its neighboring residues. Thus, this region may serve as a site to initiate the binding of the gp120 to CD4 due to its reduced entropic barrier for CD4 binding. Upon CD4 or CD4M33 binding, the depression in the mobility extends to CD4-interacting residues that neighbor Gln428. In the CD4- or CD4M33-bound states and in the ternary complex, the minimum of the depression is at Trp427, which is conserved among all primate immunodeficiency viruses and critical for CD4 binding according to mutagenesis studies [13]. This provides evidence that the initial binding may occur around the Gln428 in this region of gp120 and that a stabilization of the nearby Trp427 is likely to follow CD4 binding. The segment of gp120 that spans from residues 422 to 429 is predicted to be helical by the secondary structure prediction software, PHD [38], and indeed the conformation at residues Trp427 and Gln428 have helical character in the crystal structure of gp120 [11]. Like Trp427, Gln428 is a highly conserved residue and a Q428A mutation lowers the infection efficiency of HIV more significantly than the W427A mutations [39]. These two residues may be a critical part of gp120 for modulating the conformational changes and structural stabilization of gp120 during receptor binding.

#### Hinge sites at domain interfaces coordinate collective motions of gp120

The minima points in the global mode shapes correspond to the hinge sites that coordinate the collective motions of a molecule [40]. These sites maintain their spatial positions during the internal dynamics of the molecule. The maxima in the global modes, on the other hand, coincide with the regions that enjoy the largest conformational freedom.

The hinge sites of gp120 complexed with the D1 domain of CD4 and V_H_V_L_ domains of antibody17b, based on the combination of the first 10 slowest modes, are given in Table 1 and Figure 2C. The frequency weighted linear sums of the first 10 slow modes instead of individual modes are displayed in order to reveal the global hinge sites with a simplified approach. This approach assumes that a site acts as a hinge only if it resides on a depression point in multiple normal modes and does not enjoy high flexibility in any of the slow modes. According to the ENM analysis, all gp120 hinge sites reside on the domain interfaces between either the inner and outer domains or the bridging sheet and the other two domains. The enrichment of hinge sites at particular locations indicates a common role for these sites (Figure 2C).

**Table 1:** Hinge sites of gp120 based on gp120 ternary complex ENM calculation.

M95-K98	β1-α1	Inner-outer domain interface. β-sandwich minidomain junction
M100-E102		N-terminus of α1 helix helix
P118-K121	α1- β2	Inner domain-bridging sheet boundary
Q203-C205	β3- β4	Inner domain-bridging sheet boundary
K231-T232	*L*A	Resides on inner domain and interacts with outer domain
V255-L260	*L*B	Inner domain-outer domain boundary, Phe43 cavity
I272-V275	β10- β11	Inner domain-outer domain interface.
T283-V286	β11	Inner domain-outer domain interface. Interacts with CD4.
S364-F376	β15- α3- β16	CD4 binding loop
F382-S387	β17	Phe43 cavity.
R419-K421	β19	Outer domain-bridging sheet boundary
L452-T455	β23	Outer domain-inner domain interface. Interacts with CD4.
P470-D477	β24- α5	Inner domain-outer domain boundary. Interacts with CD4.

Hinge sites at the junctions between β strands of the bridging sheet and the outer or inner domains include regions spanning Pro118-Lys231, Gln203-Cys205 and Arg419-Lys421. These sites reside close to the positively charged surface of gp120 that mediates the interaction of the viral protein with the chemokine receptor. Also, these sites face the host cell membrane surface after CD4 binding to gp120. Among these hinge sites, Lys421 also interacts with the 17b antibody and mutational analysis has shown that this residue is important for chemokine receptor binding [22]. Interestingly, for calculations in the absence of CD4, these hinge sites are shifted such that the mobile parts are extending more into the inner and outer domains. Relative to the CD4-bound form, gp120 in CD4-free states has the Pro118-Lys121 hinge relocated to Gln114-Lys117, the Gln203-Cys205 hinge relocate to Gln209-Glu211, and the Arg419-Lys421 hinge relocate to Cys418-Ile420.

All other hinge sites reside between the inner-outer domain interfaces and align along an axis that is roughly parallel to the longitudinal axis of this domain interface. Two of these hinge sites, Thr255-Leu260 and Pro470-Asp477, are located on linkers between the outer and inner domains. Three hinge sites; Ile272-Val275, Thr283-Val286 and Pro470-Asp477 overlap with three β strands, β10, β11 and β24 respectively and reside along the outer-inner domain interface. On the other hand, loop LB (hinge-site Val255-Leu260) insinuates between β16 (hinge-site Ser364-Phe376) and β23 (hinge-site Leu452-Thr455). The β16 strand contacts β17 (hinge-site Phe382-Ser387), which in turn approaches the linker between β19 and β20 (hinge-site Arg419-Lys421). Thus, residues that are close to one another on the outer-inner domain interface, Phe43 cavity, and the bridging-sheet boundary form a network of hinges about which the rest of the protein can undergo collective motions. This indicates that structural plasticity of gp120 is coordinated by the hinges either on the linkers between the domains or aligned along the domain interfaces.

Note that the hinge site Ser364-Phe376 is a relatively long segment that starts from the CD4-binding loop and extends into the Phe43 cavity. This site becomes stabilized upon ligand binding according to the ENM analysis. In the presence of the antibody, the β16–β17 hairpin becomes completely stabilized such that this hinge site fuses with the Phe382-Ser387 hinge. It is noteworthy that mutational analysis revealed the β16–β17 hairpin to be a critical site for chemokine-receptor binding [41]. This observation confirms the importance of ligand binding on gp120, to intricately regulate the mobility and hinges on and around the Phe43 cavity and domain interfaces.

According to the ENM analysis, the Phe43 cavity is surrounded by stable hinge residues. The Val255-Leu260, Ser364-Phe376, Phe382-Phe387 and Pro470-Asp477 sites line the Phe43 cavity and are at minima on the ENM mode profile, indicating that the Phe43 cavity forms a rigid structure, where Phe43 can plug in. The Ser364-Phe376 site forms the CD4 binding loop and gets stabilized as strand β15 by CD4. Asp474 interacts with CD4 and resides on the interface between inner and outer domains. As mentioned before, Thr257 also resides on the linker between inner and outer domains and a mutation at this site reduces CD4 binding [13].

Three minima points on the slow modes profile, Asn95-Lys98, Met100-Glu102 and Lys231-Thr232 form hinge sites that coordinate the collective motions of the N - C terminal β-sandwich and also reside on the inner-outer domain interface. Mutations of various residues on this minidomain either decreased the association between gp120 and gp41 glycoproteins or resulted in structurally intact envelope glycoproteins that were inefficient in mediating syncytium formation and/or virus entry [42], despite being able to bind receptors.

### Full Atomic Normal Mode Analysis of Collective Domain Motions of HIV gp120

A better understanding of atomic details of gp120 collective domain motions, differences in the motions of gp120 in different ligation states, and the nature of the fluctuations of the residues aligning the Phe43 cavity all require a more detailed analysis such as a full atomic NMA approach. We have performed a full atomic NMA on relevant components as isolated from crystal structures. Isolated HIV gp120 alone and in its complexes with CD4D1, CD4M33 and F23 complexes are analyzed, and the motions of specific domains of gp120 are compared for these different ligation states.

**Figure 3:**
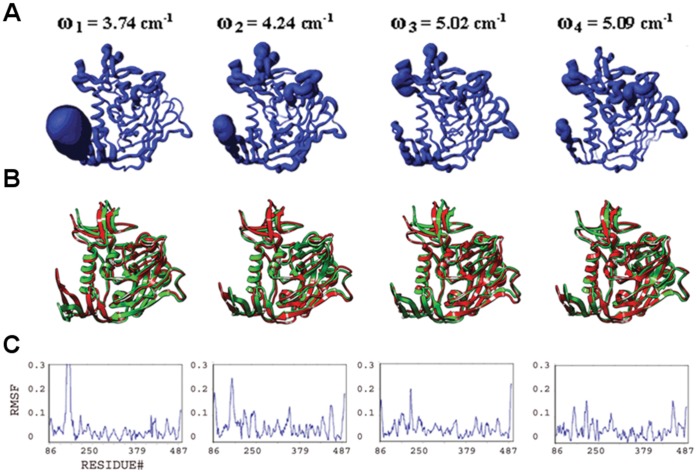
Conformational plasticity of isolated gp120. Features are shown for the four slowest modes from a full atomic normal mode analysis in a solvent shell. (**A**) Fluctuations from frequency-weighted slow modes are mapped onto worm diagrams depicting the course of the polypeptide backbone of HIV gp120. The thickness of each worm is proportional to the amplitude of the calculated fluctuations. (**B**) Superposition of conformers (red and green) generated at extremes of the fluctuations (The relative amplitude of fluctuations define the limits of the excursions on gp120; see Methods). (**C**) Alpha-carbon root-mean-square fluctuation (RMSF) plots.

In order to understand the collective motions of gp120 in absence of the CD4 receptor, the gp120 is stripped away from CD4 and the 17b Fab fragment in the crystal structures, and NMA is performed in this “isolated” state (Figure 3, Figure S1). Even though this state is possibly not highly populated in the gp120 ensemble in solution, or on the virion surface, thermodynamic analysis and antibody binding studies [8], [24] as well as recent crystal structures [43] suggest that it is one of the many states that are visited. Thus, it is crucial to calculate the atomic fluctuations in this state to understand the effect of receptor binding on the collective motions of gp120 and the role of the near native state events in the conformational conversion of gp120 to and from this state.

**Figure 4:**
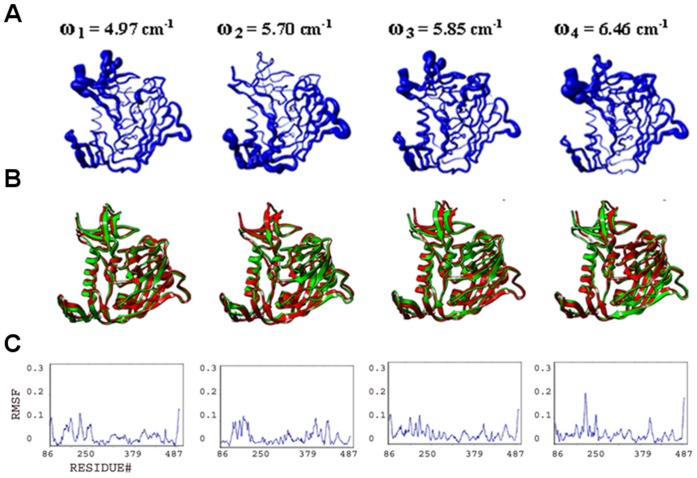
Conformational plasticity of gp120:D1. Features from the four slowest modes analyzed and displayed as in Figure 3, but for gp120 in its complex with CD4 D1. (**A**) Fluctuations mapped onto worm diagrams. (**B**) Superposition of conformers generated at extremes of the fluctuations. (**C**) Alpha-carbon RMSF plots.

**Figure 5:**
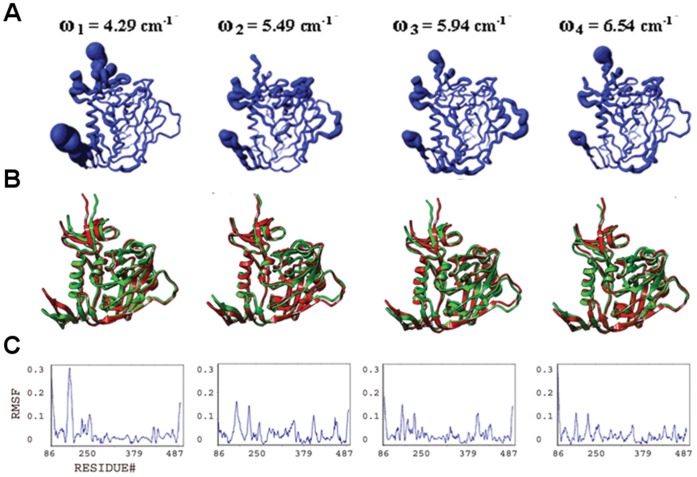
Conformational plasticity of gp120:F23. Features from the four slowest modes analyzed and displayed as in Figure 3, but for gp120 in its complex with CD4 mimetic F23. (**A**) Fluctuations mapped onto worm diagrams. (**B**) Superposition of conformers generated at extremes of the fluctuations. (**C**) Alpha-carbon RMSF plots.

**Figure 6:**
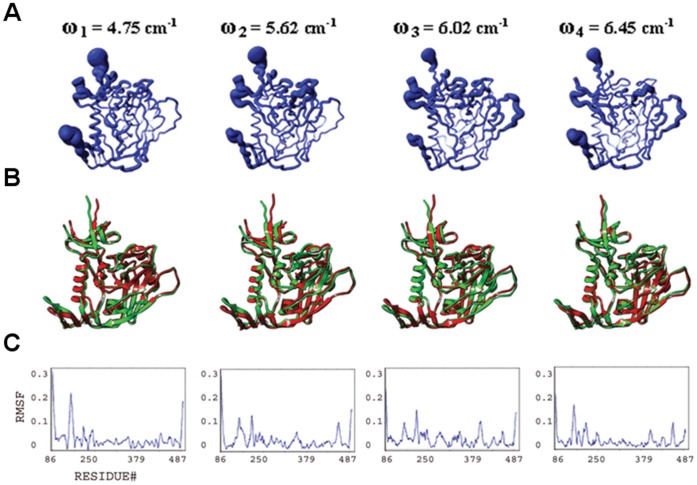
Conformational plasticity of gp120:CD4M33. Features from the four slowest modes analyzed and displayed as in Figure 3, but for gp120 in its complex with CD4 mimetic CD4M33. (**A**) Fluctuations mapped onto worm diagrams. (**B**) Superposition of conformers generated at extremes of the fluctuations. (**C**) Alpha-carbon RMSF plots.

Normal mode calculations of gp120 in the presence of CD4 are also performed. However, in order to remove the degrees of freedom arising from the potential inter-domain flexibility of CD4, the NMA is performed on the gp120:D1 complex (Figure 4, Figure S2). We analyze the fluctuations of gp120 in the presence of CD4M33 and F23 ligands together since these CD4 mimetic molecules have very similar structures and induce similar gp120 conformations. The only difference between CD4M33 and F23 is the presence of a biphenylalanine residue, which penetrates into the cavity of gp120 on CD4M33, whereas this residue is a phenylalanine in F23. The NMA of the gp120-CD4 mimetic complexes indicates that these ligands have an intermediate effect compared to CD4 on the plasticity of gp120 (Figures 5–6, Figure S3). This is consistent with both the coarse-grained ENM calculations and the titration calorimetry experiments summarized before [16]. Below, we analyze the structural plasticity of each gp120 domain and the Phe43 cavity in isolated and ligand bound states based on the slowest modes calculated with the full atomic NMA approach.

#### Collective motions and structural plasticity of the bridging sheet

he bridging sheet motions are the predominant collective motions on gp120, particularly in the isolated state, where collective motions of the β2–β3 strands dominate in modes 1 and 2 (Figure 3). In mode 1, β20–β21 strands accompany those motions but with a lower amplitude. The hinge sites Lys117 and Ser209 (on β2–β3) and Gln422 and Pro438 (on β20–β21) coordinate this collective fluctuation according to the full atomic NMA in consistence with ENM results. The direction of the fluctuation in mode 1 relates this mode to other known structures of gp120. The collective motion of the β2–β3 hairpin relocates these strands closer to the α1 helix on one end of the fluctuation. Given that the β2–β3 strands in the apo-state SIV gp120 structure have a completely different orientation and pack onto the α1 helix, this fluctuation may represent an early event in a similar conformation conversion. In the early stage of the conformational transition between a state similar to the CD4-bound one and an apo-like state, the motions of the β2–β3 hairpin, particularly around the loop region, are critical. The other extreme point of the mode-1 fluctuation relocates the bridging sheet strands away from the gp120 core. This relocation may initiate the deformation of the bridging sheet and the conformational transition to other states of gp120 (Figures 1B–C). Note that NMA of apo SIV gp120 shows high amplitude collective motions around the linkers that connect the β2–β3 to the inner domain (data not shown). This suggests two different types of initial motions taking place on two alternative states possibly to drive those states to a common intermediate. In mode 2, the fluctuations observed in the bridging sheet are confined to the tip of the β2–β3 hairpin and the amplitude is lower compared to mode 1.

The bridging sheet is significantly rigidified in the presence of CD4 according to the full atomic NMA (Figure 4). In mode 2, relatively low amplitude fluctuations of the bridging sheet are observed. Notably, the hinge sites for this motion are found on helix α1 (inner domain) and on the β16–β17 hairpin (outer domain), both of which line the Phe43 cavity. This mobile domain covers the positively charged coreceptor binding surface, which is exposed to the host cell membrane. Thus, even though CD4 binding restricts much of the bridging-sheet motion, a subtle conformational fluctuation is still observed.

The F23- and CD4M33-mimetic-bound states of gp120 display an intermediate profile between those of isolated and CD4-bound states (Figures 5–6). In mode 1, bridging-sheet hairpin β2–β3 undergoes collective motions coordinated by hinges at Val120 and Thr202 in both the F23- and CD4M33-complexed gp120. These motions are in directions similar to those seen in isolated gp120 mode 1, but amplitudes of the fluctuations are much lower for both complexes and more confined to the β-strands of the bridging sheet compared to that of isolated gp120. In mode 2, the bridging-sheet motions are similar to those in mode 1, but the amplitudes are lower for both complexes. Interestingly, in both modes, the fluctuations in F23-bound states have slightly higher amplitudes than those in CD4M33 complex. In mode 3 and mode 4, the fluctuations are confined to the tip of the β2–β3 strands in both complexes. In the CD4-mimetic-bound states, the β20–β21 strands are completely rigid.

#### Collective motions and structural plasticity of the inner domain

The inner domain consists of a two-helix, two-strand bundle at its termini-distal end and a small five-stranded β- sandwich at its termini-proximal end. Mutational studies on gp120 inner domain revealed that the β-sandwich minidomain has critical roles in subunit interactions in the gp120-gp41 trimeric complex [42]. According to the full atomic NMA, motions on this mini domain of gp120 are observed in all ligation states, and with similar amplitudes. In isolated gp120, such motions are most dominant in mode 3 spanning the residues Glu86-Asn94, Cys126-Phe233 and Lys485-Glu492 (Figure 3). Also in mode 2, the fluctuations of the inner domain fluctuations cause the opening and closing of the inner/outer domain cleft together with the fluctuations of LE and LV5 loops of the outer domain.

In the CD4-bound state, the motions of the β-sandwich become more significant compared to the rest of the molecule since the bridging sheet motions are depressed by the CD4 interactions (Figure 4). In mode 1 of gp120:D1, for example, the inner domain fluctuates under the coordination of hinge sites around Gln103, Glu211, Lys231, Val255 and Glu482 and with a rotation axis roughly parallel to the α1 helix. Mode 3 also displays collective motions of this minidomain with hinge sites around Asn95, Lys207, Cys218, Lys227, Gly235 and Leu483. This motion causes the opening and closing of the cleft between helix α1 and the minidomain. In mode 4, as in mode 1, inner-domain elements (including the loop between the β3 and β4, the β4 strand, the loop between the β7 and β8, and the C-terminal segment, which altogether form an antigenic-silent surface of the inner domain [44], fluctuate with highest amplitudes around an axis parallel to the α1 helix. The hinge sites that define the borders of the mobile residues are located around Val208, Cys228, Cys239, Arg252 and Asp474.

The normal mode profiles in F23 and CD4M33 mimetic bound states are similar to those in CD4 bound state (Figures 5–6). The only exception to this similarity is in the high amplitude fluctuations of N-terminal segments in the mimetic-bound states due to the extended conformation of this region in the mimetic bound structures. For both mimetic structures, similar to the case in CD4-bound states, a motion of the β-sandwich minidomain takes place around an axis parallel to the α1 helix in mode 1 with hinge sites around Phe233, Thr248 and Lys495. Mode 2 shapes differ from each other in the two mimetic-bound structures. Mode 2 in F23 bound state has a direction similar to mode 1 but fluctuations have lower amplitudes and hinges are located at Ile108, Glu211, Cys228 and Lys485. On the other hand, in the CD4M33-bound state the inner domain motions cause the opening/closure of the inner/outer domain cleft with hinges at Lys97, Glu211, Lys227 and Tyr486. In mode 3, a reciprocal profile of the mode 2 is observed for the F23 and CD4M33-bound states. In the CD4M33 complex, the motion takes place around an axis parallel to helix α1. In the F23 complex, the inner/outer domain cleft opens and closes as a result of the β-sandwich motion. Thus, the effect of the two peptides on gp120 is similar when mode 2 and mode 3 are considered together. However, the hinge site locations differ slightly in mode 3 compared to mode 2. In CD4M33 complex, the hinge sites are around Thr90, Ile213, Lys227 and Tyr486 in mode 3. In F23 complex, they are located around at Asn94, Lys227 and Tyr486. In mode 4, the same minidomain motion with an axis parallel to α1 helix is observed for both states.

#### Structural plasticity and collective motions of the outer domain

The outer domain is exposed to the environment and covered by a glycan shield to evade the immune responses [8], [45]. In addition to this glycan shield, the flexible loops covering the surface give additional capability for gp120 to evade antibody recognition in host environment. Fluctuations of outer domain loops are observed in normal modes in all ligation states under study. Namely, the flexible loops and the corresponding normal mode fluctuations are the LE (isolated gp120 mode 2, CD4-bound gp120 mode 4, F23 and CD4M33 bound states mode 1), LF (F23- and CD4M33-bound states mode 4), LV4 (CD4-bound state mode 4, F23- and CD4M33-bound states mode 2 and 3), LV4 (CD4-bound state mode 4, F23- and CD4M33-bound states mode 1 and mode 3), LV5 (isolated gp120 mode 4, F23- and CD4M33-bound states mode 1 and 4), V4 (CD4-bound state mode 1) and V5 (CD4-bound state mode 1).

#### Structural plasticity of the Phe43 cavity

**Figure 7:**
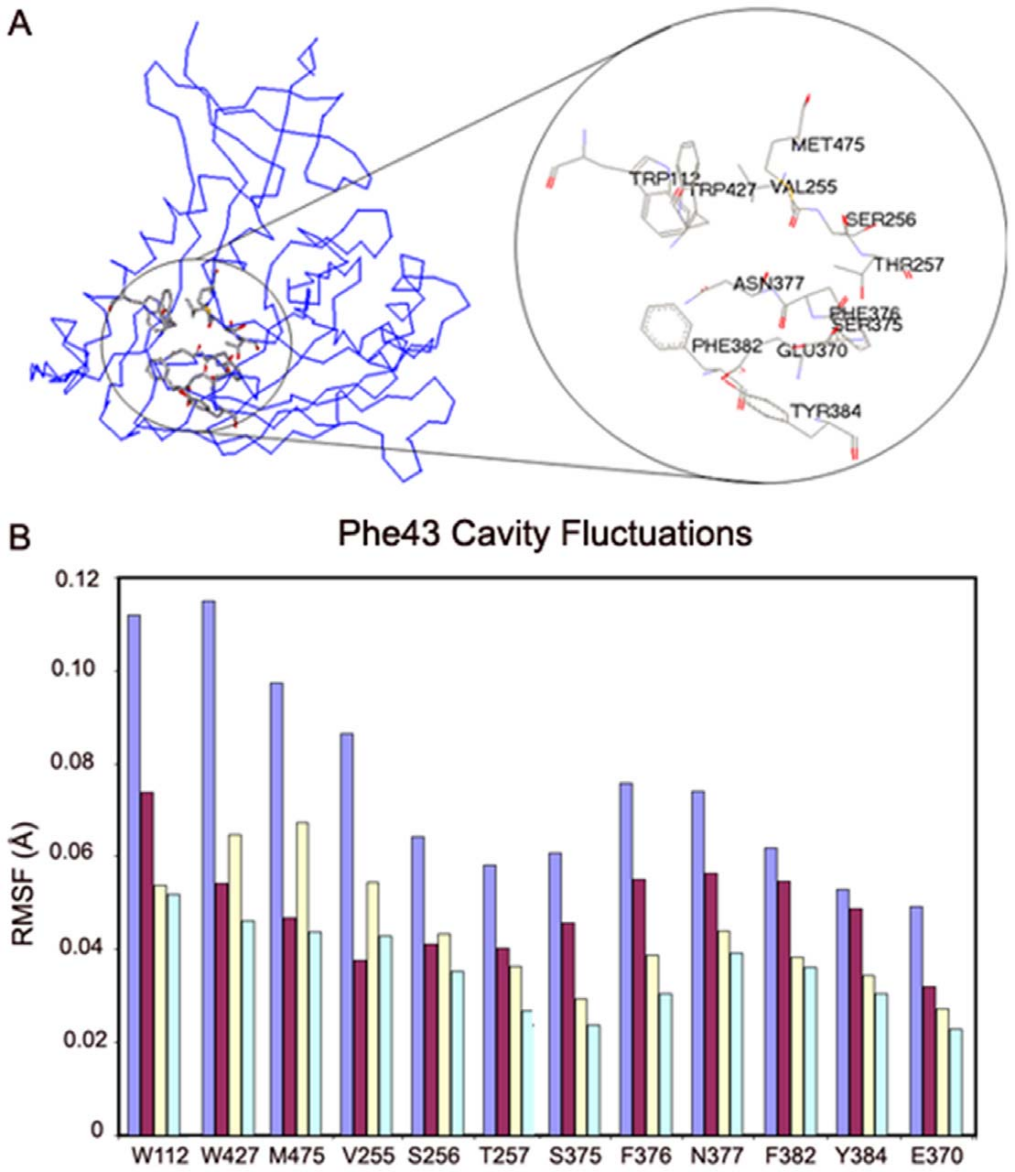
Fluctuations of Phe43 cavity residues. (**A**) Identification of residues that line the Phe43 cavity in HIV gp120. The point of view is as in Fig. 1A, but rotated clockwise about the page normal by approximately 40°. (**B**) RMS residue fluctuations of Phe43-cavity lining residues as driven by the linear combination of the 10 slowest modes from full atomic normal mode analysis. Values are shown in bars for the identified residues from isolated gp120 (light blue) and for gp120 complexed with CD4 D1 (burgundy), F23 (pale yellow) and CD4M33 (pale turquoise). Residues are placed in neighboring order, starting from W112 at upper left and moving roughly clockwise as viewed in Fig. 7A.

The Phe43 cavity is a roughly spherical cavity surrounded by conserved hydrophobic residues (Trp112, Val255, Ser256, Thr257, Glu370, Ser375, Phe376, Asn377, Phe382, Tyr384, Trp427 and Met475) (Figure 7A). In order to determine the effects of CD4 and other ligands on the Phe43 cavity, we analyzed the root-mean-square fluctuations (RMSF) of the residues that line the Phe43 cavity. The amplitudes and cross correlations of fluctuations are compared for the isolated and ligand bound states of HIV gp120.

The mass- and frequency-weighted RMSF for atoms of Phe43-cavity residues (Figure 7A) were summed over the 10 slowest modes, and the average RMSF was determined for each such residue from each of four gp120 states, isolated and complexed with CD4 D1, F23 and CD4M33. (Figure 7B, Figure S4). NMA indicates stabilization of the conserved residues around the Phe43 cavity and a general reduction in the RMSF of residues upon receptor binding. Among the cavity residues, Trp427, whose mutation impairs CD4 binding displays the most dramatic reduction in its fluctuations upon ligand binding. Other residues significantly affected are Met475, Trp112, and Val255.

Not surprisingly, the plugging of CD4M33 biphenylalanine into the cavity has a larger stabilization effect on cavity residues as compared to the phenyl groups of CD4 and F23, which only occupy the vestibule outside the cavity. Moreover, CD4M33 binding causes the cavity residues to display uniform fluctuations. The F23 binding has an effect that does not differ greatly from that of CD4. Only Trp112 is significantly more constrained and Val255, Trp427 and Met473 are less constrained in the F23 bound state.

**Figure 8:**
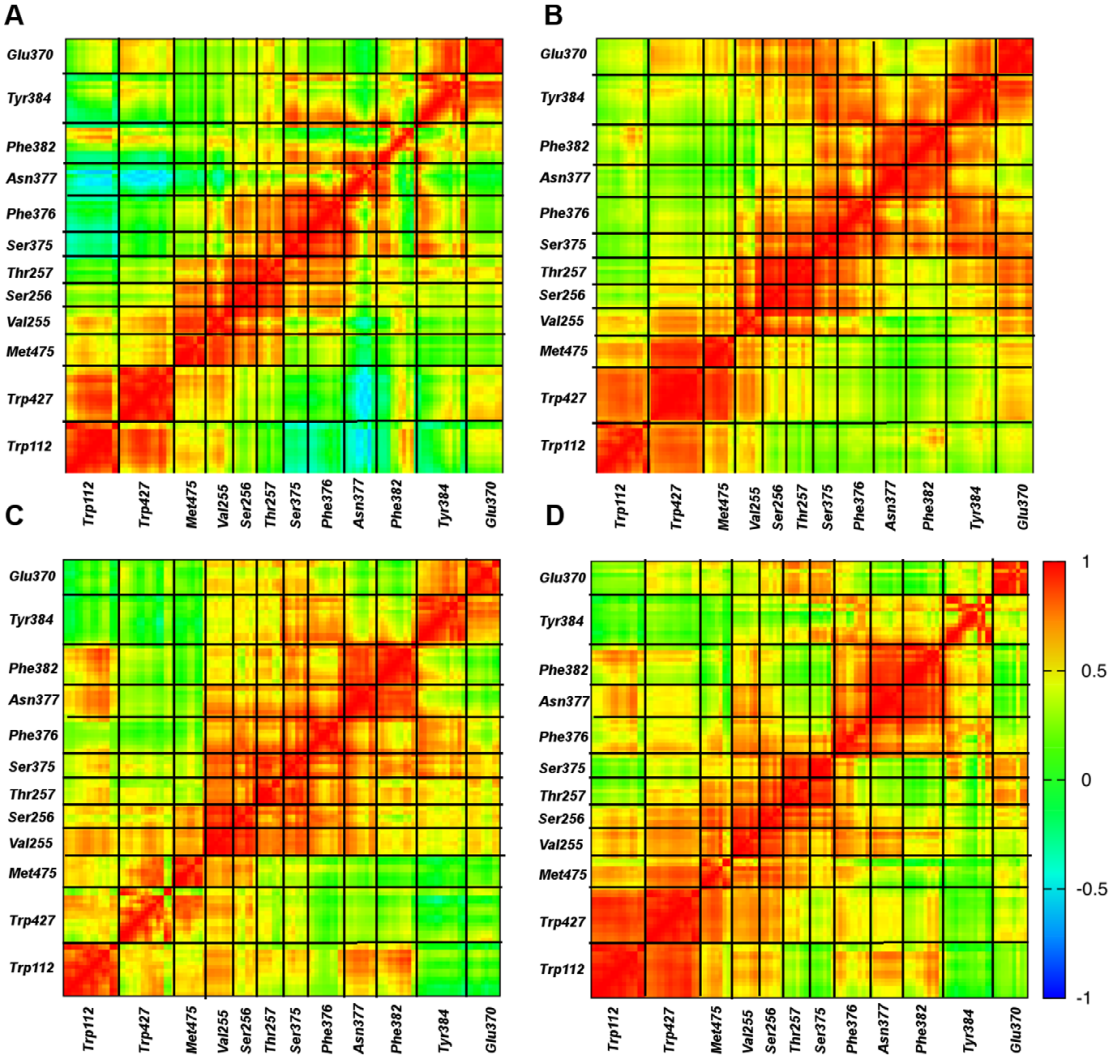
Cross correlations of fluctuations of conserved residues lining the Phe43 cavity. The cross correlation values are based on the sum of the 10 slowest modes. (**A**) gp120. (**B**) gp120:CD4(D1). (**C**) gp120:F23. (**D**) gp120:CD4M33. Specific residues are identified in the same order as in Fig. 7B.

The cavity residues fluctuate with higher amplitudes in the absence of any ligand. However, the directionality and the cooperative character of these motions also play a role in the function of the cavity in inducing and stabilizing the CD4-bound state of gp120. Thus, the cross-correlation of the atomic fluctuations were calculated for the 10 slowest modes of motion and given in the contour maps for different ligation states of HIV gp120 (Figure 8).

Correlation of atomic motions is an important aspect of the functional motions of proteins such as enzyme-substrate recognition [46] and allosteric regulation [47]. We analyze the cross correlations in residue fluctuations around the receptor binding sites in gp120 to determine whether similar phenomena exist in gp120. NMA enables the calculation of cross correlations between different atoms by the expression [48]

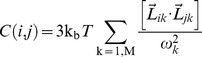
(1)where **L_ik_** is the eigenvector related to the k_th_ normal mode and acting on atom i with frequency ω_k_. M is the number of normal modes included in the calculation, k_b_ is the Boltzmann constant, and T is the temperature. In the isolated gp120, no correlation peak is observed within the cavity residues indicating random fluctuations in the cavity in spite of high fluctuation amplitudes. Such random motions may possibly reflect the initiation of the cavity deformation in the absence of any ligand.

Contrary to the isolated state, the Phe43 cavity residues have highly correlated fluctuations in the presence of CD4 (Figure 8B). CD4 binding not only reduces the amplitude of fluctuations but also induces a more cooperative character in the cavity residues even when they are not in immediate proximity of each other. Particularly, fluctuations of Glu370 and Tyr384 become highly correlated with much of the rest of the cavity upon CD4 binding. In fact, the correlation map indicates that the cavity residues form two groups that are partially isolated from each other in terms of fluctuation cross-correlations. One group comprises residues Trp112, Trp427, Met475 and to some extent Val255, while the other group is formed by Val255, Ser256, Thr257, Glu370, Ser375, Phe376, Asn377, Phe382, Tyr384.

In the F23 bound state, the cross-correlations of the cavity fluctuations are similar to those in the CD4-bound state (Figure 8C). However, important differences are also observed. For example, even though the cooperative regions stay similar, the levels of correlation are lower in the presence of F23. In addition, the correlation between Trp112 and Trp427 are limited to the side chain atoms in the F23 bound state. Glu370 and Tyr384 lose their cooperative character with other residues that displayed high cross correlations in the CD4-bound state. All of these differences lead a lessening of the general bipartite character of the cavity motions observed in the CD4 state.

In the presence of CD4M33, the fluctuation cross-correlation profile is less ordered compared to CD4 and F23. This near random behavior of cavity atoms, marked by extended low cross-correlations, may be due to the cavity filling by the biphenylalanine of CD4M33. As seen in the isolated state, no correlation peaks are observed in the off-diagonal regions on Figure 8D (i.e. high cross-correlations are limited to atoms in close contact). This supports the notion that CD4M33 binding uniformly represses the motions of the cavity residues without inducing the profound effects of CD4. It is also notable that whereas fluctuations of Asn377 are negatively correlated with those of Trp112 and Trp427, as to some extent are those of neighboring Ser375 and Phe376; such cross-cavity correlations, including those with Phe382, are enhanced positively in the ligand complexes, especially so for CD4M33.

### VAMM Analysis of Conformational Transitions of HIV gp120

**Figure 9:**
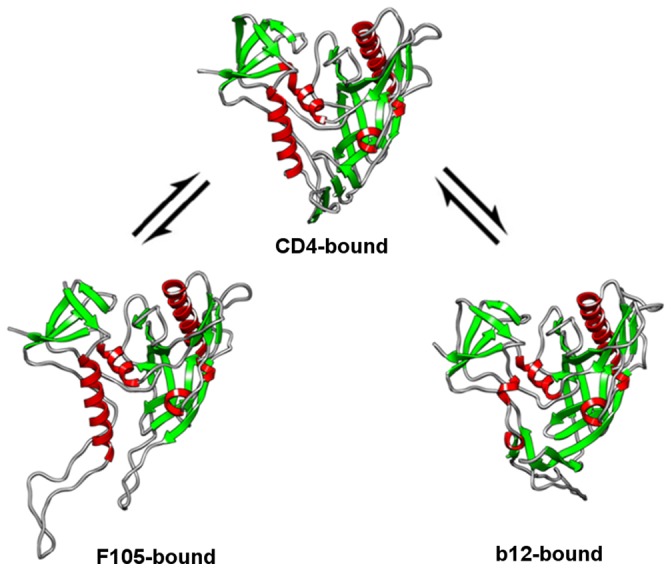
Conformational equilibria between CD4-, F105- and b12-bound conformations of gp120.

Current information on the distinct conformational states of gp120 based on crystallography [11], [17], thermodynamic experiments [8] and analysis of antibody binding to cross-linked gp120 molecules [24] suggest that distinct gp120 conformations are connected to each other with large-scale conformational transitions (Figure 9). Conformational states that display similar properties with CD4 and b12-bound states have been shown to exist in solution in the absence of any ligand [43]. Here, we calculate the transition pathways of gp120 between experimentally determined states of gp120 (i.e. CD4-bound, b12-bound and F105-bound) using the VAMM force field and algorithm.

#### Conformation of gp120 varies in CD4 and antibody bound states

Large conformational differences are observed between structures of gp120 in CD4-bound and antibody bound states. The RMSD between the CD4 and b12-bound states of gp120 is 12.5 Å, whereas the RMSD is 8.3 Å between the CD4 and F105-bound states. Between the CD4 and b12-bound states, the conformational differences involve complete rearrangement of bridging sheet domain, a helix to coil transition on the inner domain of gp120 and other minor rearrangements around the loops, inner-outer domain cleft and variable loops. The V1-V2 stem of the b12-bound gp120 is disordered in the crystal structure and was computationally modeled (See methods) for VAMM calculations. The differences between the CD4 and F105-bound states are mainly around the bridging sheet, with minor deviations in variable loops. In the F105-bound state, both β2–β3 and β20–β21 stems of the bridging sheet are deformed. Unlike in the b12-bound state, the α1 helix remains intact in the F105-bound state. In the gp120:F105 complex crystal structure, the LV4 is disordered and the loop connecting β2–β3 is replaced by a short Gly-Gly-Ser sequence. Both loops were computationally modeled (See methods).

#### Transition pathway intermediates converge to a common intermediate state

**Figure 10:**
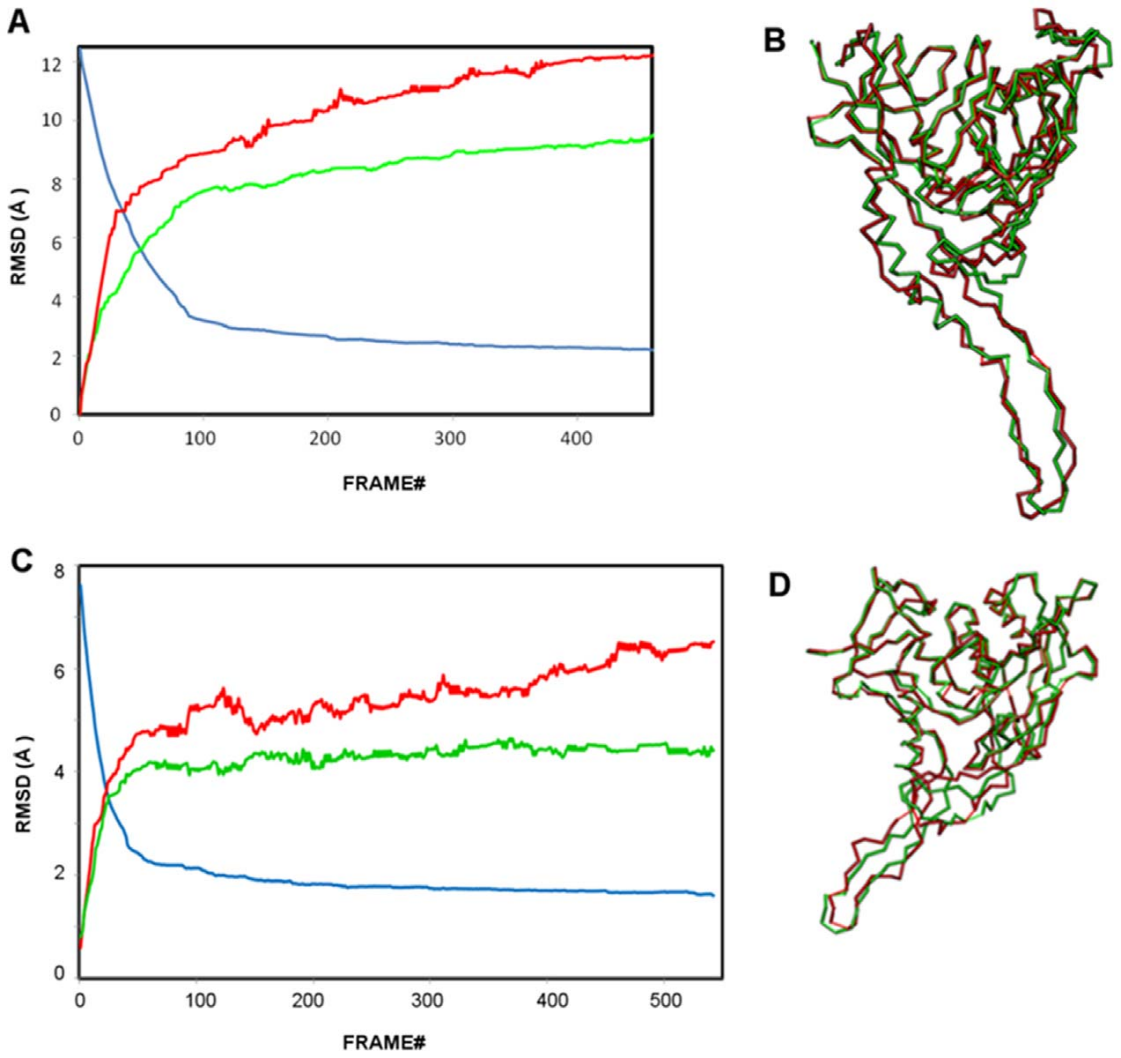
The convergence of gp120 conformational transitions. (**A**) RMSD profiles for the gp120 conformational transition between CD4- and b12-bound states. Blue curve: The RMSD between the intermediate states generated from CD4- and b12-bound states. Red curve: RMSD between the initial b12-bound state and the intermediates generated from this state. Green curve: RMSD between the initial CD4-bound state and the intermediates generated from this state. (**B**) Superposition of Cα backbones of terminal intermediate structures originating from CD4-bound (green) and b12-bound (red) states. (**C**) RMSD profiles for the gp120 conformational transition between CD4- and F105-bound states. Colors are as for (A) but apply to F105 rather than b12. (**D**) Superposition of Cα backbones of terminal intermediate states originating from CD4-bound (green) and F105-bound (red) states.

The transition pathways between CD4- and antibody-bound states (i.e. b12 and F105) are computed using the VAMM algorithm. The RMSD profiles for intermediates generated in these pathways between the CD4- and the b12- and F105-bound states are shown in Figures 10A and C, respectively. For both cases, the algorithm converges to two terminal intermediate states, which are generated from two end states and are highly similar to each other.

The gp120 conformational transition between CD4-bound and b12-bound states is computed for 977 VAMM iterations until the two terminal point conformations converge to conformational states that differ from each other for only 2 Å. In 460 steps, the two states converge to terminal intermediates that deviate from each other with an RMSD of ∼2.2 Å. After this point, the transition pathway enters a plateau phase and no significant structural rearrangement takes place between iterations 460 (convergence: 2.2 Å) to 977 (convergence: 2.0 Å). That is why we do not analyze the plateau phase trajectory beyond the 460^th^ iteration. In fact, in 183 steps, the pathway reaches to 2.7 Å convergence from an initial 12.5 Å RMSD, which indicates a rapid transition of gp120 structures to a relatively similar common state and a following slow phase that involves minor structural rearrangements. The RMSD profile for the computed transition is given in Figure 10A.

The conformational transition between CD4-bound and F105-bound states is computed for 542 VAMM iterations until the two terminal point conformations converge to conformational states that differ from each other by only 1.6 Å. In only 115 steps, the pathway reaches to 2.0 Å convergence from an initial 8.3 Å RMSD, which again indicates a rapid transition state followed by slow phase that involves minor structural rearrangements. As expected, the transition pathway for F105-bound gp120 converged more efficiently than for the b12-bound case since this pathway does not involve the helix-to-coil transition around the α1 helix. The RMSD profile for the computed transition is given in Figure 10C.

In Figure 10B, the terminal intermediates generated from CD4-bound and b12-bound are superimposed. Although, the majority of the structures align well, there is a series of regions with significant deviations, possibly due to the very high energetic costs to complete such transitions. In the terminal intermediate states, the major differences are observed between residues that cover the C-terminal half of the α1 helix and the V1/V2 stem of the deformed bridging sheet. In both regions, the two terminal intermediate states have the exact same orientation but secondary structure transition is required to fully link the two states. In Figure 10D, the terminal intermediates generated from CD4-bound and F105-bound are superimposed. In the two terminal intermediates, minor deviations between β2–β3 and β20–β21 strands are observed. The β-strand to coil transformation in these regions prevents further convergence of the computed structures to completely identical intermediate states. Rest of the molecules has very similar structures.

#### Antibody-bound states have high conformational variability

The intermediates generated from the antibody-bound states deviate from the initial structure more than those generated from the CD4-bound state. The terminal intermediate state generated from the b12-bound has an RMSD of 12.23 Å from the initial b12-bound state, whereas the corresponding value from the CD4-bound state is 9.48 Å. This suggests that the b12-bound state enjoy a larger structural plasticity compared to the CD4-bound state, consistent with the relatively low binding entropy of b12 antibody to gp120 compared to sCD4. Similarly, the terminal state generated from F105 state has a 6.51 Å RMSD from its initial state, while this value is 4.4 Å for the other terminal state. The more extended conformational transitions observed in antibody bound states are mainly due to the larger structural flexibility enjoyed by the V1/V2 stem of the deformed bridging sheet.

#### Movements during conformational transitions of gp120

**Figure 11:**
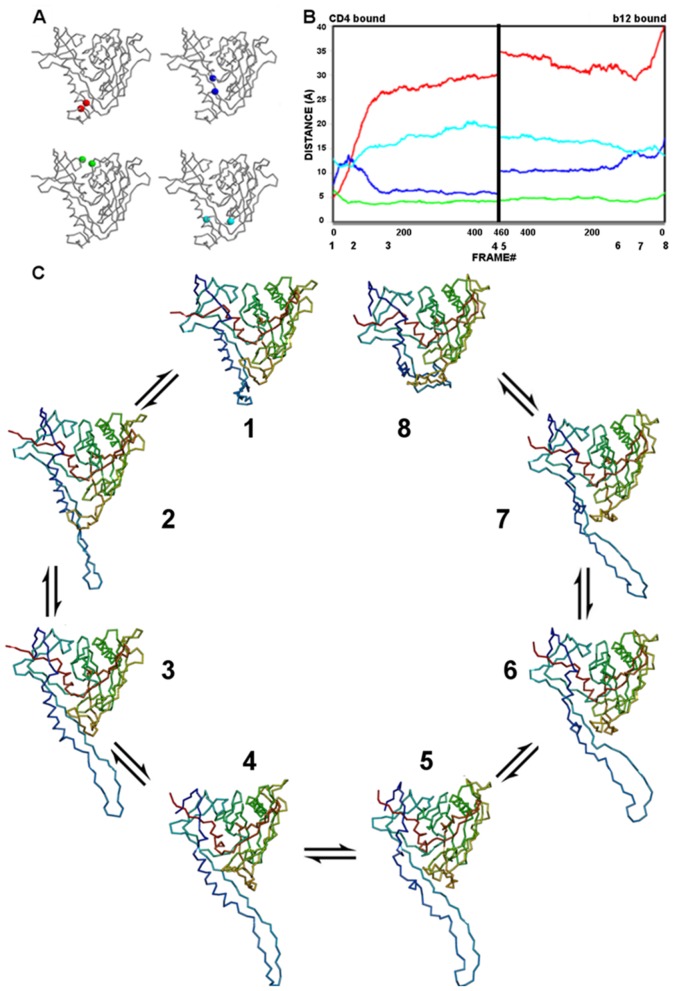
The gp120 transition pathway between CD4- and b12-bound states. (**A**) Monitor cues mapped onto Cα-backbone drawings of gp120 in the CD4-bound state. Stability of the bridging sheet is monitored by the distance between Thr123 and Gly431 (red); opening of the vestibule of the Phe43 cavity is monitored by the distance between Trp427 and Met475 (blue); the inner/outer domain cleft is monitored by the distance between Lys231 and Glu267 (green); and the integrity of the Phe43 cavity is monitored by the distance between Trp112 and Phe382 (cyan). (**B**) The progression of monitor distances, colored as in **A**, during the gp120 trajectory. Numbers below the x-axis mark the positions of structures visualized in panel **C**. (**C**) The identified gp120 transition intermediates along the trajectory starting from the CD4-bound state (1) and ending at the b12-bound state (8). Structures are drawn as Cα backbones colored spectrally from N (blue) to C (red).

**Figure 12:**
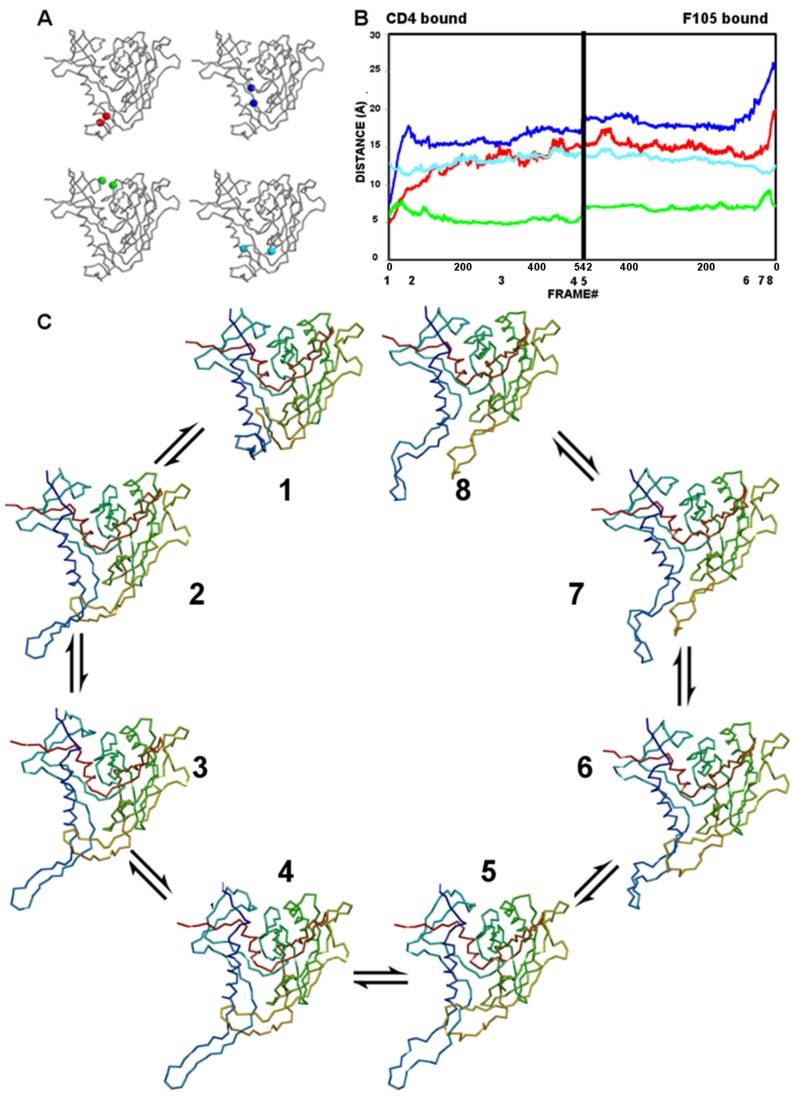
The gp120 transition pathway between CD4- and F105-bound states. (**A)** Monitor cues as in Figure 11**A**. (**B**) The progression of monitor distances, colored as in **A**, during the gp120 trajectory. Numbers below the x-axis mark the positions of structures visualized in panel **C**. (**C**) The identified gp120 transition intermediates along the trajectory starting from the CD4-bound state (1) and ending at the F105-bound state (8). Structures are drawn as Cα backbones colored spectrally from N (blue) to C (red).

The conformational transitions of gp120 between antibody- and CD4-bound states are characterized by a combination of motions in the bridging sheet, the Phe43 cavity, the helix-to-coil transition of the α1 helix, and the inner-domain β-sandwich. In both conformational transitions, the dominant motion observed is the rearrangement of the β2–β3 strands to generate an extended conformation of gp120. The characteristics of the computed transition pathways are monitored by the progression of distances in residue probes located on critical regions of the molecule (Figures 11–12), and the changes monitored by these probes are described in the subsections below:

#### Bridging-sheet transitions

The deformation of the bridging sheet is the major event observed along both of the transition pathways computed between CD4-bound state and antibody-bound states. It is monitored by measuring the distance between Thr123 on the β3 strand and the Gly431 on the β21 strand. In the CD4-bound state, which has an intact bridging sheet, the distance between Thr123 and Gly431 is 4.8 Å (Figure 11A). In both F105 and b12-bound states, the bridging sheet is completely deformed and the Thr123-Gly431 distances are 40.6 Å and 19.9 Å, respectively.

During the conformational transition from the CD4-bound to b12-bound state, the monitor distance increases up to 26.5 Å in 132 steps, then enters a plateau phase and slowly increases to 29.3 Å (Figure 11B). This is consistent with the view that a rapid deformation of the bridging sheet is observed in the absence of CD4 binding as suggested by titration calorimetry experiments. On the other hand, the bridging-sheet strands are already separated from each other in the b12-bound state. In the early stage of the transition originating from the b12-bound state, this distance reduces to 28.9 Å from 40.6 Å in 126 steps and then gradually increases to 34.8 Å, which is close to the maximum distance observed in intermediate states originated from the CD4-bound state.

A similar bridging-sheet transition is observed on going from the CD4-bound to the F105-bound state (Figure 12B). In the CD4-bound state, the bridging sheet deforms, the β2–β3 strands gradually protrude from the core gp120, and the Thr123-Gly431 probe distance increases from 4.8 Å to 14.0 Å in 200 steps and then remains mostly unchanged along the remaining trajectory. In the F105-bound state, the two stems of the bridging sheet rapidly come to close proximity in 80 VAMM iterations and form a transition state, in which the bridging sheet is partially deformed and the probe distance is 13.4 Å. After this point, the probe distance slightly increases to a maximum value of 17.5 Å in the late phase of the transition.

For both computed transition pathways, the motions of the bridging sheet beta strands, particularly those of β2–β3 strands dominate the transition pathways observed between gp120 states. These motions lead to the complete deformation of the bridging sheet in these antibody complexes, where the β2–β3 strands assume an extended conformation protruding from the core gp120.

#### Vestibule opening to the Phe43 cavity

Understanding the evolution of the Phe43 cavity during the conformational transition is critical to understanding how this cavity affects other conformational changes. One feature of the cavity is its vestibule that CD4 Phe43 occupies outside the cavity. The vestibule opening is monitored by the distance between the two critical residues located on the entrance of the cavity. These residues are Trp427 on the β20–β21 hairpin and Met475 on the inner/outer domain linker between β24 and α5. In the CD4-bound state, the initial Trp427-Met475 distance is 7.4 Å (Figure11A), whereas these distances are 17.6 Å and 25.5 Å in b12-bound and F105-bound states, respectively.

For the CD4- and b12-bound states, the transition pathway trajectory reveals that the cavity samples two different phases on the trajectory (Figure 11B). In the initial stage of the transition pathway from the CD4-bound state, the cavity vestibule opens while the probe distance increases to 13.7 Å in 45 VAMM iterations. In the following phase, the distance reduces to 5.9 Å at step 148 and becomes relatively stable in following VAMM iterations. On the other end of the trajectory, Trp427 and Met475 distance reduces to 13.0 Å after 35 steps from the 17.6 Å observed in initial b12-bound state. After this initial vestibule closure, the vestibule opens slightly (probe distance = 14.5 Å) until step 83 and slowly recloses to a probe distance of 10.40 Å.

The Phe43 cavity vestibule has a more open configuration in the F105-bound state with an initial probe distance of 25.5 Å. The nature of the transition pathway between the CD4- and F105-bound states reflects this initial open conformation (Figure 12B). In the trajectory initiated from F105-bound state, the vestibule rapidly closes in 107 steps to 17.5 Å from 25.5 Å, and it remains stable for the rest of the transition pathway. Intermediates that are generated from the CD4-bound state show a transition profile in going toward the F105-bound state as in the transition to the b12-bound state. In the first phase the probe distance increases from 7.4 Å to 17.8 Å in 55 steps and in the second phase the vestibule partially closes, such that the probe distance is reduced to 15.7 Å in step 91. However, unlike the case in transition to the b12-bound state, the vestibule does not assume a fully closed conformation and remains more open compared to its initial state. In the late phase of the transition, the probe distance increases from 15.5 Å to 17.1 Å for intermediates generated from CD4-bound state and from 17.7 to 19.1 Å for intermediates generated from F105-bound state. Thus, the vestibule fluctuates between partially open and partially closed states along the trajectory between CD4- and F105-bound states.

#### Integrity of the Phe43 cavity

The integrity of the Phe43 cavity is monitored with the distance between the Trp112 and Phe382 residues that line opposite walls of the cavity. Along both of the computed transition pathways, the relative motions of these residues are negatively correlated with those of Phe43 vestibule probes Trp427 and Met475. In the CD4-bound state, the distance between the Trp112-Phe382 cavity probes is 13.0 Å (Figure 11A). The probe distances are 12.1 Å and 13.6 Å for b12- and F105-bound states, respectively.

For the CD4- and b12-bound states, the probe distance reduces to 11.1 Å from 13.0 Å in CD4-bound state at step 45, while the Phe43 cavity vestibule has an open configuration (Figure 11A). After this point, the distance between the Trp112 and Phe382 slowly increases up to 20.5 Å, while the Phe43 cavity closes. The current trajectory suggests a mechanism whereby the two opposing walls of the cavity separate from each other simultaneously with the closure of the vestibule opening by the β20-β21 hairpin. The transition pathway generated from the b12-bound state also displays a similar anti-correlated vestibule opening vs. cavity stability profile with smaller changes in the Trp112-Phe382 distance. The probe distance first increases to 15.7 Å from 12.1 Å, then slightly reduces to 14.4 Å and gradually increases to 17.5 Å.

The trajectory generated between the CD4- and F105-bound states reveals a very similar profile although this cavity stays more stable along the trajectory (Figure 12B). The Trp112-Phe382 distance fluctuates between 11.3 Å and 14.6 Å along the overall trajectory. As in the other computed pathway, the distance between the Trp112-Phe382 is anti-correlated with that of Trp427-Met475.

#### Inner-outer domain cleft

The evolution of the distance between residues Lys231 and Glu265 monitors the relative movements of inner and outer domains. In the CD4-bound state, the initial distance is 5.8 Å (Figure 11A). In the b12- and F105-bound states, the initial distances are 6.2 Å and 7.2 Å respectively.

In the transition pathway between CD4- and b12-bound states, the inner-outer domain movements have lower amplitudes compared to changes in bridging-sheet and Phe43-cavity monitors (Figure 11B). The Lys231-Glu265 distance has a minimum value of 3.9 Å (step 42) for intermediates generated from CD4-bound state. In the late phase of the transition, the cleft slightly opens to a probe distance of 4.5 Å. A similar pattern is observed for the intermediate states generated from the b12-bound state. It initially reduces down to 4.2 Å indicating a slight domain closure at step 87 and then opens to assume a probe distance of 5.0 Å. The inner-outer domain interface stays stable during the late phase of the transition. The observed transition pattern suggests that the inner domain fluctuates between closed and open states with low amplitudes.

The inner-outer domain distance fluctuates with higher amplitudes in the transition trajectory between CD4 and F105-bound states (Figure 12B). In the trajectory generated from CD4-bound state, the inner-outer domain distance rapidly increases to 8.0 Å at step 38 from its initial value of 5.8 Å. After the peak value, the cleft closes back and the probe distance assumes a minimum probe distance at 4.8 Å. In parallel, the probe distance increases up to 9.3 Å at step 18 from an initial value of 7.2 Å on the other end of the trajectory. After this peak value, the cleft closes back with a minimum probe distance of 6.5 Å. At both sites of the trajectory, the inner outer domain cleft displays minor fluctuations during the late phase of the transition pathway.

## Discussion

In parallel with the crystallographic, biophysical and biochemical studies, the computational analysis of gp120 suggests that there is a direct link between the conformational transitions and structural plasticity of gp120 and its function. Thus, gp120 dynamics have biological significance.

The most prominently dynamic domain of gp120 is the bridging sheet. In our isotropic elastic network analysis, the overall bridging sheet is highly mobile in the absence of any ligand. The computational analysis shows a direct link to conformational masking mechanisms of gp120 to evade immune responses. Furthermore, the analysis gives a detailed picture of this molecular mechanism. Binding of ligands to the isolated gp120 gradually stabilizes the bridging sheet in parallel with the titration calorimetry experiments. The lack of directional information in ENM results makes it impossible to understand the atomic details of this stabilization. A full atomic NMA confirms the stabilization of gp120 bridging sheet with ligand binding.

he full atomic NMA, which gives information on the near native fluctuations, suggests that all four strands of the bridging sheet move together in the same direction; however, the VAMM-based conformational transition computation reveals a two-phased mechanism based on the evolution of the Trp427-Met475 distance that monitors the vestibule opening (Figure 11 & Figure 12). Trp427 resides on the β20–β21 hairpin and its position relative to Met475 reflects the position of this hairpin relative to the gp120 core as well as the vestibule opening on Phe43. The bridging-sheet strands move out as a body in the early phase of the transition pathway, consistent with motions obtained from full atomic NMA. This opens the cavity and disrupts the interactions between the critical sites of the cavity in the open state. In the later phase, however, the β20–β21 strands change direction to cap the Phe43 cavity, while the β2–β3 strands keep their extended conformation, protruding from the gp120 core. The Phe43 cavity becomes less accessible as a result, but the CD4 binding residues on the β20–β21 hairpin are still accessible to interactions by ligands. The b12-bound state monotonically assumes the closed cavity conformation from a more open state in its early phase. In the later stages of the transition, the cavity reopens. Furthermore, it was shown experimentally that deletion of β20–β21 residues Ile424 to Lys432 enhances the binding of b12 and F105 antibodies 60 to 100 fold and this enhancement is explained by reduction of steric hindrance around the CD4 binding site in the absence of the β20–β21 loop [49]. This observation on deletion of β20–β21 strands is consistent with the computed closed-conformation of the β20–β21 strands in the transition.

The Trp112-Phe382 distance that monitors cavity integrity is negatively correlated with the Trp427-Met475 distance that monitor vestibule opening in both computed transition pathways. This negative correlation implies that cavity openings and closings take place simultaneously with cavity deformations. Thus, these results suggest that conformational masking mechanisms involve not only the deformation of the bridging sheet and motions of the β2–β3 strands but also an equilibrium of β20–β21 conformations and cavity stability that is related to the opening and closing of the Phe43 cavity (Figure 11B). The anti-correlated motions of the cavity residues along the transition pathways suggest a complementary, and possibly mutually exclusive, immune evasion mechanism of steric and entropic barriers to antibody binding and neutralization.

In parallel with these motions, the comparison of local fluctuation on the Phe43 cavity in different ligation states reveals a mechanism of cavity stabilization by CD4 through reduction of fluctuations in this cavity and also through the synchronization of those restricted motions. Similar symmetric motions have been shown to play an important role in determining the enzymatic specificity of the α-lytic protease [46]. In gp120, it has been shown by mutational studies that the structure of the Phe43 cavity plays a critical role in determining the conformation of the overall structure. For example, the S375W mutation induces a CD4-like conformation in the absence of CD4 [15]. Similarly, our computational analyses suggest that limited but synchronized motions in cavity-lining residues contribute in an important way to Phe43 cavity stabilization.

The current analyses of gp120 dynamics rely on the use of monomeric gp120 core structures in complexes with various ligands (i.e. receptors, receptor mimetic molecules and antibodies). Such analyses successfully explain the domain motions and transitions of gp120 in isolation and the effect of various ligands on gp120 plasticity. However, gp120 exists in a trimeric form on the viral surface as associated with the gp41 trimer, and it is cooperative conformational changes of this trimeric gp120:gp41 complex that drive the viral entry process. A computational analysis of gp120 in this trimeric form is required for elucidating the molecular mechanism of viral entry in atomic detail. Our computational tools and systematic approach provide a framework for such analyses, which await availability of atomic-level trimer structures.

HIV gp120 operates in a diverse spatiotemporal spectrum, and conformational dynamics play important roles in both viral entry into host cells and conformational masking of HIV to evade the human immune system. Thus, any therapeutic strategy that targets HIV gp120 should extend beyond the analysis of static structural information and benefit from systematic and comprehensive analyses of gp120 dynamics.

## Methods

### Protein Structures

The crystal structure of gp120 core from the YU2 primary isolate in complex with a two- domain fragment of CD4 and the antigen-binding fragment of a human antibody at 2.9 Å resolution (pdb id: 1G9N) was used for the ENM and NMA calculations. The crystal structures of gp120 from YU2 isolate in complex with CD4M33 at 2.75 Å resolution (pdb id: 1YYL) and F23 at 2.2 Å resolution (pdb id: 1YYM) were used to analyze the dynamic properties of gp120 when it was in complex with CD4 mimicking peptides. The V4 loop of gp120 in the CD4M33 and F23 bound state was modeled using the RAPPER loop modeling program [50] since this loop was not modeled into the electron density map of the original crystal structures.

The gp120 from HXBc2 in the b12 antibody-bound state (PDB ID: 2NY7) and the gp120 in complex with CD4 and 17b antibodies (pdb ID: 1G9M) were used in transition pathway calculations. MODELLER software [51] was used to model the 20-residue long V1–V2 stem, which was disordered in the crystal structure of the gp120:b12 antibody complex. In order to determine the correct orientation of the loop, V1–V2 stem was modeled in a gp120:b12 crystal lattice with space group P6_5_22 and unit cell of dimensions as in the crystal structure. All atoms within 60 Å of Val120 and Ala204, including symmetry mates, were included so as to ascertain an allowed conformation for the V1–V2 stem in the crystal lattice. The disulfide bridge between Cys126 and Cys196 was included in the model as an initial restraint recognized by the MODELLER software. The gp120 structure with the modeled V1–V2 stem was used in VAMM-based transition state calculations. The structure of loop LV4 for the CD4-bound state was modeled by first patching in loop LV4 from the YU2 gp120 structure (PDB ID = 1G9N) and then by refining the resulting structure using the MODELLER software.

The gp120 structure from YU2 in the F105-bound state (PDB ID: 3HI1) was used in VAMM calculations. A series of loop and homology modeling studies were carried out before VAMM calculations. First, a homology model of HXBc2 was constructed using the YU2 structure as the template (sequence identity = 89%). Next the disordered LV4 and the GAG sequence that replaced LV3 was modeled by using the MODELLER software. Finally, the truncated segment of 9 residues in the β2–β3 hairpin loop was patched from the CD4-bound state and further refined using the MODELLER software.

### Isotropic Elastic Network Analysis (ENM)

ENM inspects the local packing density and coordination of each amino acid to determine its range of motions available in the folded state [32]. The simplicity of ENM is a result of two main assumptions of the model: (i) the “coarse-grained” approach, according to which each amino acid is represented only by its α-carbon atom and the whole protein is viewed as an elastic network made of α-carbon nodes and (ii) a simple harmonic potential with a single spring constant for each residue type. The network topology is defined by a Kirchhoff matrix Γ, in which the off-diagonal elements are −1 if α carbons of the residues i and j are closer than a cut-off distance (7.0 Å) and diagonal element values are the negative sum of all off-diagonal elements in their column. The autocorrelation of residue i, which yields the mean square fluctuation of this particular residue, is found from:
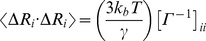
(2)where *k_b_* is the Boltzmann constant, *T* is the absolute temperature, and [Γ ^−1^]*_ij_* is the *ij^th^* element of the inverse of Γ.

Information about global dynamics is acquired by decomposing the motions into a series of eigenmodes and concentrating on the modes at the slowest end of the spectrum. The autocorrelation [ΔR_i_•ΔR_i_]_k_ contributed by the kth mode is found from
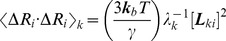
(3)where **L** *_k_* is the *k*th eigenvector of Γ and λ*_k_* is the *k*th eigenvalue.

### Protein Solvation, Equilibration and Energy Minimization

**Figure 13:**
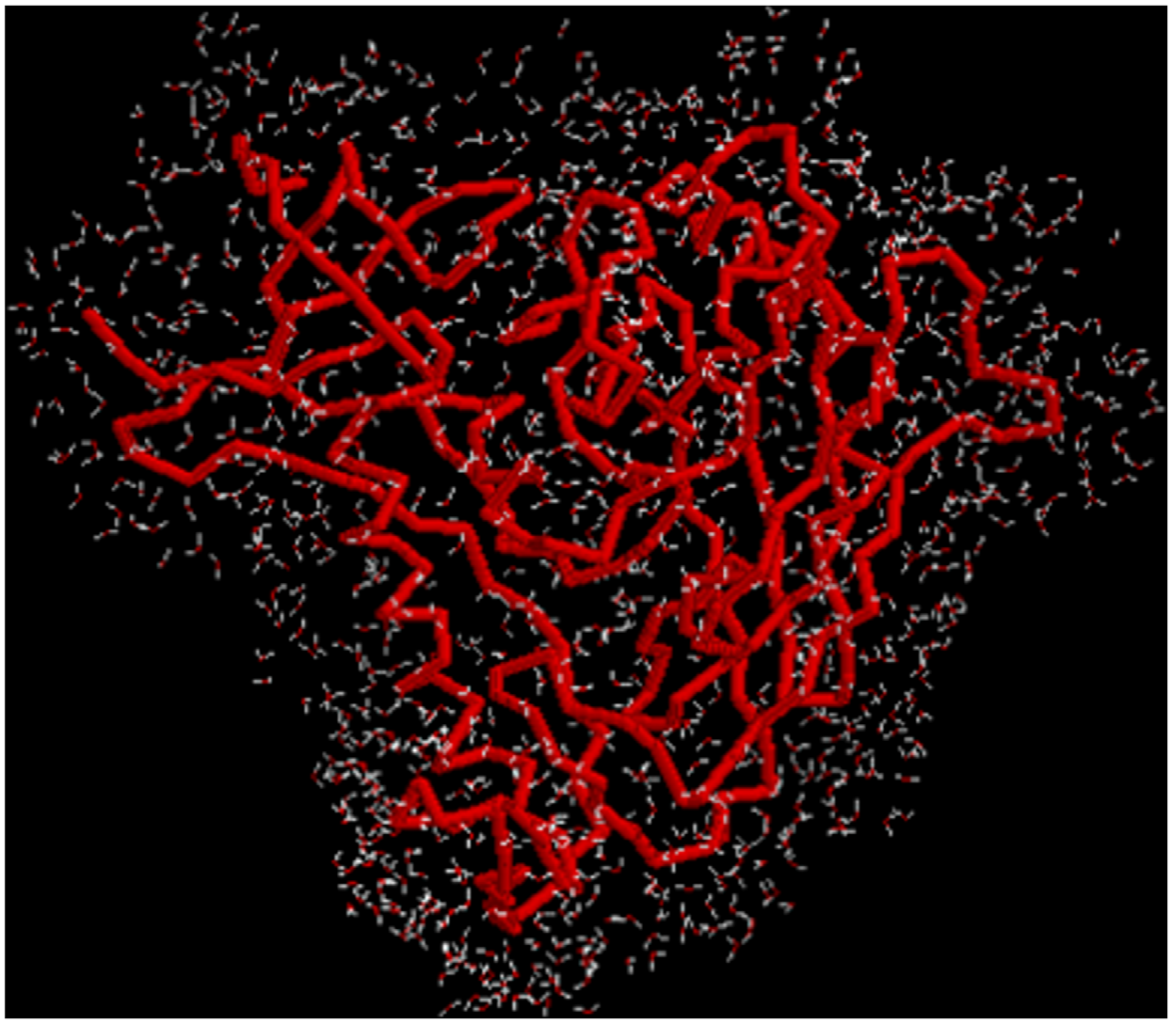
The solvent shell for molecular dynamic simulation of HIV gp120. The isolated gp120 molecule is shown as a backbone worm and the 1150 TIP3P water molecules are shown as oxygen ball (red) and O-H bond sticks (white).

NMA of macromolecules requires fully minimized structures of those macromolecules in order to obtain positive eigenvalues and real frequencies from the normal mode decomposition. Use of a vacuum system for energy minimizations prior to NMA may lead to significant deviations from the crystal structures, especially when force fields like CHARMM 22, which has specifically been designed for energy calculations of proteins in water solvent is used for minimizations in vacuum. In order to reduce the deviations in the structures prior to NMA, a solvent shell was constructed around the proteins and all minimizations and NMA calculations were performed on proteins in this solvent shell (Figure 13). The CHARMM simulation package and all atom CHARMM 22 molecular force field were used in all equilibration runs, minimizations and normal mode calculations [36]. The parameters for the biphenyl ring on biphenylalanine of CD4M33 were adapted from reference [52]. First, each protein structure was minimized for 500 steepest descent steps to remove any residual bad contacts or strains in the structure. Next, protein complexes were solvated by a 5.0-Å aqueous solvent shell prepared by immersing the complex in a large sphere of TIP3P water [35] and deleting any water molecule having its oxygen atom at a distance either less than 2.8 Å or more than 5 Å from non-hydrogen atoms of the solute. A fully flexible TIP3P model was used for all minimizations and normal-mode calculations. The number of total water molecules varied between 1150 (isolated gp120) and 1552 (gp120:D1). The nonbonding interaction parameters were set such that the electrostatic interaction was shifted to zero at 12 Å and the van der Waals interaction was switched off from 10 Å to 13 Å. A constant dielectric constant (ε = 1) was set since an explicit solvent model was used. A distance-dependent dielectric constant (ε = 4r) was adopted for minimizations in vacuum in order to mimic the effect of solvent.

In order to relax the water molecules, a 50-ps equilibrium molecular dynamics simulation was run at 300 K for the solvent shell, keeping the protein rigid. A 0.5-fs time step and the leap-frog method numerical integrator were used for the molecular dynamics simulations. The initial velocities were assigned by randomly sampling a Gaussian distribution at 300 K. Before the equilibrium dynamics, the water shell was heated from 0 to 300 K during a 5-ps simulation, increasing the temperature by an increment of 6 K every 0.1 ps. For the rest of the 45-ps simulation, the temperature was kept constant at 300(±10) K.

After equilibrating the solvent shell, the protein structures were energy minimized (Table 2) as in [46]. First, 100 steps each of steepest descent minimizations were performed using harmonic constraints of 200, 100, 50, 20, 10, and 5 kcal/mol Å^2^ on all non-hydrogen atoms. This was followed by 500 steps of steepest-descent minimization with no harmonic constraints in order to further relax the protein system. Finally, Adapted Basis Newton Raphson (ABNR) minimization was applied until the rms gradient fell below 10^−6 ^kcal/mol Å (Table 2).

**Table 2:** The RMSD between minimized and crystal structures of gp120.

	Vacuum		Solvent Shell	
	Main chain	All atoms	Main chain	All atoms
gp120	1.59	0.8	0.81	0.89
Gp120:D1	2.01	2.13	1.05	1.20
Gp120:CD4M33	2.44	2.55	0.67	0.75
Gp120:F23	2.24	2.36	0.81	0.88

### Normal Mode Analysis

The diagonalization in mixed basis (DIMB) method [53] implemented in the CHARMM simulation package was used to calculate the low frequency normal modes from the mass-weighted Hessian Matrix. The DIMB method does repetitive reduced-basis diagonalizations and uses less memory compared to the conventional diagonalization but yields exactly the same eigenvalues and eigenvectors as for the conventional diagonalization methods. Only the first 250 normal modes, whose frequency values were less than 50 cm^−1^, were computed with an eigenvalue and eigenvector accuracy tolerance level of 0.05.

The domain motions along each normal mode were analyzed by adding and subtracting the normalized eigenvector of that normal mode to the minimized structure. The normal modes were visualized such that each deformed conformation and the minimized structure have a RMSD of 10.0 Å divided by the frequency of the particular normal mode. Resulting RMSD values between extreme deformed conformations varied between 2.56 Å (isolated gp120, mode 1) and 1.30 Å (gp120:F23, mode 4) for the first four normal modes that are analyzed.

### VAMM Transition Pathway Calculation

The transition pathways between the CD4-bound and antibody-bound states of gp120 were calculated with the VAMM transition algorithm and force field. The VAMM algorithm yields a transition pathway between two alternative conformations, A (e.g. CD4-bound gp120) and B (e.g. b12-bound gp120), by moving each structure toward the other in iterations of moves directed at each step along the normal mode of greatest engagement with its target structure. Before the VAMM analysis, both states were energy minimized to an initial gradient of 0.1 kcal/mol Å using a gradient descent minimization algorithm implemented in VAMM software. During the transition pathway calculation, the secondary structure information for intermediate states was updated at every 0.1 Å deviation from previous states along the trajectory using the DSSP algorithm [54]. Twenty slow modes were monitored at every NMA step to select the one with the highest involvement coefficient. The step sizes of moves were scaled as
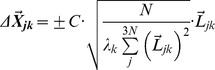
(4)where C is a constant chosen as 0.04, λ_k_ is the eigenvalue of mode k, L_jk_ is the eigenvector for mode k acting at atom j, and ± refers to the alternative directions for this mode. The energy of the intermediate states was minimized to an energy gradient of 0.3 kcal/mol Å, when the energy gradient exceeded 1.5 kcal/mol Å.

## Supporting Information

Figure S1 **Structural plasticity of gp120 around the isolated state.** The atomic fluctuations are calculated using full atomic NMA in a solvent shell (see main text). Fluctuations of gp120 in **(A)** mode 1, (**B)** mode 2, **(C)** mode 3, **(D)** mode 4. View as “slide show” to visualize fluctuation animations.
(PPTX)

Figure S2 **Structural plasticity of gp120 around the CD4-bound state.** The atomic fluctuations are calculated using full atomic NMA in a solvent shell (see main text). Computation is performed with the gp120:D1 (i.e., the Domain 1 of CD4, which contains all gp120 interacting sites.) Fluctuations of CD4-bound gp120 in **(A)** mode 1, (**B)** mode 2, **(C)** mode 3, **(D)** mode 4. Only gp120 is displayed. View as “slide show” to visualize fluctuation animations.
(PPTX)

Figure S3 **Structural plasticity of gp120 around the F23 and CD4M33-bound states.** The atomic fluctuations are calculated using full atomic NMA in a solvent shell (see main text). Fluctuations of F23 and CD4M33-bound gp120 in **(A)** mode 1, (**B)** mode 2, **(C)** mode 3, **(D)** mode 4. Only gp120 is displayed. View as “slide show” to visualize fluctuation animations.
(PPTX)

Figure S4 **Structural plasticity of Phe43 Cavity.** The atomic fluctuations are calculated using full atomic NMA in a solvent shell (see main text.). The gp120 cavity fluctuations for isolated, CD4, F23 and CD4M33-bound states in **(A)** mode 1, (**B)** mode 2, **(C)** mode 3, **(D)** mode 4. View as “slide show” to visualize fluctuation animations.
(PPTX)
